# Role of Energy Metabolism in the Brown Fat Gene Program

**DOI:** 10.3389/fendo.2015.00104

**Published:** 2015-06-30

**Authors:** Minwoo Nam, Marcus P. Cooper

**Affiliations:** ^1^Division of Cardiovascular Medicine, Department of Medicine, University of Massachusetts Medical School, Worcester, MA, USA

**Keywords:** brown fat, mitochondria, respiratory capacity, thermogenesis, brown fat gene program

## Abstract

In murine and human brown adipose tissue (BAT), mitochondria are powerful generators of heat that safely metabolize fat, a feature that has great promise in the fight against obesity and diabetes. Recent studies suggest that the actions of mitochondria extend beyond their conventional role as generators of heat. There is mounting evidence that impaired mitochondrial respiratory capacity is accompanied by attenuated expression of *Ucp1* and other BAT-selective genes, implying that mitochondria exert transcriptional control over the brown fat gene program. In this review, we discuss the current understanding of brown fat mitochondria, their potential role in transcriptional control of the brown fat gene program, and potential strategies to treat obesity in humans by leveraging thermogenesis in brown adipocytes.

## Introduction

Brown fat is composed of thermogenic adipocytes that convert chemical energy to heat. Found in homeothermic animals such as mammals, brown fat protects against cold stress. In neonates, brown fat plays a critical role in thermoregulation. The thermogenic capacity of brown fat is attributed to abundant mitochondria, which dissipates heat via uncoupling (1).

There are two types of thermogenic adipocytes: classical brown adipocytes and beige/brite adipocytes. Classical brown adipocytes exhibit constitutive thermogenic capacity with large numbers of mitochondria. Developmentally programed brown fat, such as the rodent interscapular depot, consists of these cells. Another type of thermogenic adipocytes is found in white fat. These cells are recruited by thermogenic stimuli and in turn display comparable thermogenic capacity to classical brown adipocytes. Due to these properties, they have been named beige/brite (brown-in-white) adipocytes (1, 2).

Brown fat has unique genetic signatures that support its thermogenic function. Uncoupling protein 1 (*Ucp1*), a key thermogenic protein, is highly expressed in brown fat. Cell death-inducing DFFA-like effector (*Cidea*), a modulator of UCP1, and Type II iodothyronine deiodinase (*Dio2*), an enzyme that converts T4 to active T3 within the tissue locally, are among major thermogenic genes (3–5). Fatty acid oxidation (FAO) genes and electron transport chain (ETC) subunit genes are also enriched (6). This transcriptional signature is in part determined by PR domain containing 16 (*Prdm16*), a determinant of brown fat, and peroxisome proliferator-activated receptor gamma coactivator *1*-alpha (*Ppargc1a*), a key coactivator of peroxisome proliferator-activated receptor alpha (*Ppara*) and gamma (*Pparg*) (7, 8). The transcriptional network of the brown fat genes have been reviewed elsewhere (9). Since many of the brown fat-selective genes regulate thermogenesis, hereafter “the thermogenic gene program” and “the brown fat gene program” are reciprocally used.

Mitochondria play a central role in providing an energetic basis for thermogenesis in brown fat. Brown fat mitochondria fulfill their duty as cellular powerhouses in the non-stimulated state. In sympathetically stimulated brown fat, UCP1 is rapidly activated and uncouples electron transit and ATP production. Instead, the chemical energy stored in the proton gradient is dissipated as heat (10). Interestingly, several studies have demonstrated that impaired mitochondrial respiratory capacity is accompanied by attenuated expression of *Ucp1* and other brown adipose tissue (BAT)-selective genes in classical brown adipocytes (11, 12). In addition to a conventional role as generators of heat, this suggests that brown fat mitochondria have an unappreciated role in regulating genes involved in thermogenesis depending on the status of respiratory capacity. Critical evaluation of these studies will broaden our thoughts on mitochondria in brown fat. This review summarizes the features of brown fat mitochondria and discusses their potential role in transcriptional control of the brown fat gene program and its therapeutic implications in humans.

## Features of Brown Fat Mitochondria

### Morphological features

Brown fat mitochondria are dynamic organelles that meet the thermogenic needs of the organism by regulating their number and networking as well as their biochemical and ultrastructural profile. Acute cold exposure (or activation with norepinephrine) immediately promotes mitochondrial fission, an event that precedes and augments thermogenesis (13). Brown fat mitochondria exhibit unique morphological features. Notably, brown fat mitochondria are enlarged and densely packed with respiratory units, resulting in dense cristae (14). The morphological features of brown fat also differ across gender with females having larger mitochondria and denser cristae (15). The density of brown fat mitochondria is among the highest of any tissue (16). Even so, chronic cold exposure increases mitochondrial mass even further. This is mediated by catecholamines via β-adrenergic signaling, which increases PGC-1α, a transcriptional coactivator that induces ERRα and NRF-1, culminating in increased mitochondrial mass (17–19).

### Biochemical features

Early biochemical and functional studies on brown fat mitochondria in rodents revealed high cellular respiration but low ATP synthase activity (20). This implied that pathway involving proton leakage must underlie the basis of thermogenesis. Biochemical studies identified that UCP1 constitutes the molecular basis for enhanced proton leak (21). Interestingly, in larger mammals such as lambs the abundance of ATP synthase is higher (22), presumably because larger animals are less dependent on non-shivering thermogenesis due to their smaller surface-to-volume ratio and due to their increased capacity for shivering.

Long-chain free fatty acids (LCFAs) activate UCP1, while purine nucleotides inhibit UCP1 (10). Intuitively, mobilization of free fatty acids via β-adrenergic activation is coupled to UCP1 activation and heat production. Fedorenko et al. (23) reported that UCP1 does not exhibit constitutive proton transport activity. Instead, there is obligatory binding of LCFAs to UCP1, a process that transfers protons associated with LCFA into the matrix via a conformational change of UCP1. LCFAs can also overcome inhibition by purine nucleotides. This is important because in the basal state, there is no lipolysis and inhibition of UCP1 by purine nucleotides will promote coupled ATP-generating respiration.

In theory, uncoupling should de-energize brown fat mitochondria, culminating in an ATP crisis. ATP, however, is required for the activation of fatty acids during uncoupling. Brown fat mitochondria circumvent this by increasing glycolysis as well as the TCA cycle. Arsenite, an inhibitor of pyruvate dehydrogenase complex (PDC) and α-ketoglutarate dehydrogenase, depleted ATP in norepinephrine-stimulated brown adipocytes, implying that the TCA cycle is critical for maintaining ATP during thermogenesis (24). Although succinyl-CoA synthetase primarily generates GTP, this TCA enzyme complex can also generate ATP, a process which may explain how the TCA cycle is critical for maintaining ATP in brown fat (discussed further in Proteomical Features). Glycolysis is an important source of ATP, too. Notably, hexokinase activity increases fourfold in cold acclimated rats, achieving glycolytic activity similar to liver (25).

### Proteomical features

Mass spectrometric analysis of brown fat mitochondria has revealed striking proteomic difference compared with white fat mitochondria (26). In fact, the proteomic profile of brown fat mitochondria was most similar to that of skeletal muscle. Compared with white fat mitochondria, there was an enrichment of catabolic pathways including ETC, TCA cycle, and fatty acid metabolism in brown fat mitochondria. Complexes I–IV are present at higher levels, whereas complex V is present at lower levels, a pattern favorable for thermogenesis. There is robust expression of enzymes involved in the TCA cycle–ADP-forming succinyl-CoA synthetase β subunit (A-SCS-β), pyruvate dehydrogenase kinase 4 (PDK4), and pyruvate dehydrogenase phosphatase regulatory subunit (PDPr). SCS converts succinyl-CoA to succinate. This reaction is coupled to the formation of ATP or GTP, which is determined by two different β subunits, ADP-forming and GDP-forming. In mouse, rat, and human, metabolically active tissues, such as brain and heart, express high levels of ADP-forming subunits compared with GDP-forming subunits (27). Likewise, brown fat mitochondria may preferentially use A-SCS-β to supply ATP, a feature that matches a role of substrate-level phosphorylation in stimulated brown adipocytes. During cold exposure, lipid uptake and lipogenesis replenish fat stores that have been oxidized (28). Control of lipogenesis during cold exposure is complex and partly regulated by pyruvate metabolism (29, 30). Pyruvate can be targeted for complete oxidation by converting it into acetyl-CoA via the enzymatic action of PDC (31). Alternatively, inhibition of PDC by PDK4 diverts pyruvate into glycerol, which is the backbone for free fatty acid (FFA) esterification (31). An enzymatic complex consisting of PDPr counteracts the action of PDK4, and thus, targets pyruvate for complete oxidation (32, 33). In summary, opposing regulation by PDK4 and PDP may play a critical role in whether or not the brown adipocyte uses pyruvate for lipogenesis (PDK4-mediated) or complete oxidation (PDPr-mediated).

Fatty acids serve as major substrates for thermogenesis and they activate UCP1 (10). In brown fat, there is a high expression of enzymes involved in FAO, including short-, medium-, long-chain acyl-CoA dehydrogenases and 3-ketoacyl-CoA thiolase (26). Long-chain fatty acids require a carnitine palmitoyltransferase 1B (CPT1B)-mediated carnitine shuttle for oxidation in mitochondria. Supporting a role for robust oxidative capacity in brown fat, CPT1B is 50-fold higher in brown fat compared with white fat (26). Brown fat may also exhibit metabolic flexibility in fuel utilization. Highly expressed in brown fat, acetyl-CoA synthetase 2-like (ACSS1) permits oxidation of ketone bodies during starvation (26, 34, 35). Indeed, activity of ketone body oxidizing enzymes in brown fat parallels that of the heart (36).

## Role of Energy Metabolism in the Brown Fat Gene Program

Although manipulating regulators of mitochondrial biogenesis, such as PGC-1 coactivators and ERRα, affects *Ucp1* expression, those factors are also known to directly regulate transcription of respiratory subunits (37), making it challenging to delineate a direct relationship between respiratory capacity and the thermogenic gene program *per se*. Here, we discuss approaches to directly manipulate mitochondrial respiratory capacity and the attendant effects on the brown fat genes, which are summarized in Table 1.

**Table 1:** **Effects of manipulating mitochondrial respiratory capacity on the brown fat gene program**.

	Model	Respiratory capacity	Mitochondrial mass	Brown fat morphology	Brown fat genes	Other	Reference
	COX7RP KO	↓10–20% in O_2_ consumption	n/a	Hypertrophic Pale brown	↓75% in *Ucp1;* ↓*Dio2*, *Elovl3*	Normal *Erra*, *Nrf1*, *Tfam*	Ikeda et al. (12)
	TFAM FKO (Fabp4-Cre)	↓40–60% in complex I and IV; ↑70% in complex II (BAT); ↑30% in O_2_ consumption (BAT); ↑80% in state3 OCR (BAT mt)	↑20% in citrate synthase activity	Normal	Normal	↓DIO ↓Insulin resistance	Vernochet et al. (38)
Mouse	TFAM FKO (Adipoq-Cre)	↓40–80% in complex I and IV (BAT)	↑80% in citrate synthase activity	Whitening	Normal	↓DIO ↑Insulin resistance Lipodystrophy and inflammation in WAT	Vernochet et al. (39)
	CRIF1 FKO (Fabp4-Cre)	↓OXPHOS subunits protein	↓Mitochondrial abundance ↑Mitochondrial size	Smaller size	Unaltered UCP1	Defective WAT postnatal death at week 3	Ryu et al. (40)
	LSD1 Tg (IWAT)	↑*Nrf1, Cpt1b, Cox8b* complex II and IV	↑Mitochondrial abundance	↑Beige/brite adipocytes in IWAT	↑*Prdm16, Ppargc1a, Ucp1*	↓DIO ↓Insulin resistance	Duteil et al. (41)

Cell	LRPPRC KD in brown adipocytes	↓20% in O_2_ consumption			↓40–75% in *Ucp1, Cidea, Cox7a1*		Cooper et al. 2008 (11)

*↓, Decrease; ↑, increase; KO, knockout; Tg, transgenic; FKO, fat-specific KO; KD, knockdown; mt, mitochondria; BAT, brown adipose tissue; IWAT, inguinal white adipose tissue; DIO, diet-induced obesity; n/a, not assessed*.

### Leucine-rich pentatricopeptide repeat containing motif (LRPPRC; also called leucine-rich protein 130 kDa, LRP130)

A potential role of mitochondrial respiratory capacity in the brown fat gene program was reported in a study using LRPPRC-deficient brown adipocytes (11). LRPPRC was originally identified as a causal protein in a rare neurological disorder called Leigh Syndrome French Canadian variant (42). Initial studies using human fibroblasts identified defects in cytochrome *c* oxidase deficiency; however, later studies using mouse models revealed that LRPPRC affects the expression of all mitochondrially encoded subunits of the ETC but their differential effects on respiratory complex activity related to cell type (43–46). Brown adipocytes with depleted LRPPRC were notable for impaired oxygen consumption but intact mitochondrial density and PGC-1 coactivators, indicating a specific impairment of respiratory capacity without altering mitochondrial biogenesis (11).

LRPPRC-deficient brown adipocytes had a marked reduction in brown fat-selective genes, including *Ucp1* and *Cidea*, suggesting a link between respiratory capacity and a basal expression of certain brown fat genes (11). While LRRPRC is weakly expressed in the nucleus, recent data show that the majority of LRPPRC is localized to mitochondria and regulates mtDNA-encoded transcripts across various species, suggesting that a nuclear role of LRPPRC in the regulation of brown fat genes may be modest and that the predominant effect is mediated by impaired cellular respiration (44, 47–49). In addition, cAMP-mediated induction of *Ucp1* was unaffected in LRPPRC-deficient brown adipocytes (11). Given that PGC-1α is responsible for this cAMP effect, it is less likely that LRPPRC is an essential part of PGC-1α coactivator complexes necessary for *Ucp1* expression. Furthermore, in human fibroblasts, deficiency of LRPPRC did not affect expression of PGC-1α target genes (50), implying that signals secondary to impaired cellular respiration, not reduced nuclear expression of LRPPRC, are likely important.

### Cytochrome C oxidase subunit VIIa polypeptide 2-like (COX7RP)

Recent work using COX7RP knockout mice provides direct evidence for the role of respiratory capacity in the brown fat gene program (12). In this study, COX7RP was identified as a novel assembly factor for respiratory chain supercomplexes in mitochondria. ETC complexes are known to form supercomplexes, consisting mainly of complex I, III, and IV (so-called respirasome), which enhances respiratory activity (51). With reduced oxygen consumption at a whole-body level, COX7RP KO mice revealed hypertrophic and pale brown fat, generally indicative of defective brown fat. More importantly, *Ucp1* was severely reduced in this dysmorphic brown fat. Microarray analysis also showed a downregulation of several brown fat-enriched genes including *Dio2* and *Elovl3*. Expression of PGC-1 coactivators was decreased but their downstream targets, such as *Erra*, *Nrf1*, and *Tfam*, were unaltered, implying no significant impact on the PGC-1 coactivator network. All together, this study strongly suggests that respiratory capacity dictates a retrograde signaling from mitochondria to the nucleus regulating the brown fat gene program.

### Transcription factor A, mitochondrial (TFAM)

One approach to genetically manipulate respiratory capacity is to target components of the basal transcriptional machinery of mitochondrial DNA (mtDNA). Among them is TFAM, a key player in mtDNA transcription and maintenance (52). Fabp4-Cre-driven loss of TFAM led to diminished respiratory activity and a drop in mtDNA copy number in brown and white fat (38). Paradoxically, in those mice, brown fat showed enhanced respiratory capacity as evidenced by increased oxygen consumption, FAO, and citrate synthase activity. Although reduced in weight, this brown fat had normal expression of brown fat genes. Similarly, brown fat markers were intact in brown fat with Adipoq-Cre-driven loss of TFAM, which was accompanied by increased citrate synthase activity (39). Therefore, it is likely that the unaltered brown fat gene program in TFAM-deficient fat is ascribed to a compensatory increase in respiratory capacity. Although not decisive, observations from adipose-specific TFAM knockout mice imply that whole-cell respiratory capacity is monitored by an innate sensor to dictate the brown fat gene program.

### CR6-interacting factor 1 (CRIF1)

CRIF1 is a mitochondrial protein that controls the translation and insertion of mitochondrially encoded respiratory subunits into the inner membrane (53). The activities of Complexes I, III, and IV are abrogated by CRIF1 deficiency in mouse embryonic fibroblasts (53). The severity of impaired respiratory capacity by ablation of CRIF1 is evident in brain-specific and cardiac muscle-specific knockout mice in which severe neurodegeneration and premature death develop, respectively (53, 54). Adipose-specific CRIF1 knockout mice (driven by Fabp4-Cre) show a developmental defect in white fat, reduced body weight, and postnatal death at week 3 (40). Brown fat in these mice is smaller in size; however, histology is unremarkable and UCP1 expression is normal. Because mice die by 3 weeks of age, it was not possible to assess the chronic effect of respiratory defects in brown fat. Although mice with Adipoq-Cre-driven deletion of CRIF1 were viable, data regarding the brown fat genes were not shown (40). Even so, these data could suggest that impaired mitochondrial function does not influence brown adipocyte development. Future studies will be necessary to address if mitochondrial function is critical for the maintenance of the brown fat program. Finally, as mentioned for TFAM, the method by which cellular respiration is disrupted may have differential effects on mitochondrial signaling and subsequent transcriptional events in the nucleus.

### LSD1 (lysine-specific demethylase 1)

LSD1 demethylates mono- and di-methylated lysines (particularly lysine 4 and 9 of histone H3) via the cofactor flavin adenosine dinucleotide (FAD) (55). Ubiquitously expressed, LSD1 is essential for embryogenesis and tissue-specific differentiation (56). In the study by Duteil et al. (41), LSD1 was newly identified as a cold-, and β3-adrenergic signaling-inducible protein in mouse white fat. Ectopic expression of LSD1 further revealed that it was sufficient to induce respiratory capacity through nuclear respiratory factor 1 (NRF1) in white adipose cell lines. In addition, there was an activation of the brown fat gene program including *Prdm16*, *Ppargc1a*, and *Ucp1* in LSD1-overexpressing white adipocytes. LSD1 transgenic mice confirmed these *in vitro* findings. Interestingly, this browning effect was more robust in subcutaneous white fat where beige adipocytes reside. However, in brown fat of LSD1 transgenic mice, respiratory activity was modestly increased and there were no significant changes in the brown fat markers. These data suggest that augmented respiratory capacity may promote browning by stimulating beige adipocytes.

## Therapeutic Implications

There is an association between impaired brown fat function and various animal models of obesity and diabetes, including *ob/ob* mice, *db/db* mice, SHR/N-*cp* rats, and high-fat/high sucrose (HFHS)-fed mice (57–61). A similar association has been reported for human obesity (62–64). In the aforementioned murine models of obesity and diabetes, there is “de-browning” of brown fat, characterized by concomitant reduction in UCP1 protein, and respiratory complex activities (40, 53–56). Similar to classical brown adipocytes, beige adipocytes exhibit this type of de-browning in mice fed a high-fat diet, implying a shared mechanism for all types of thermogenic adipocytes (65). As detailed earlier, several models and systems have established a causal relationship between mitochondrial respiratory capacity and transcription of genes involved in thermogenesis. Impaired respiration in certain models of obesity and diabetes may explain impaired transcription of the thermogenic genes. In the future, altering respiratory capacity may prove promising in restoring brown fat function in certain forms of obesity and diabetes. Even if there are no effects on transcriptional programs involved in thermogenesis, augmenting respiratory capacity *per se* could still effectively protect against obesity and diabetes by way of increased respiratory capacity, which in turn would increase thermogenic capacity.

In humans and mice, cold exposure and β3-adrenergic agonists activate brown fat to promote energy expenditure (10, 66–68). Many adrenergic receptor (AR) agonists, including pan-adrenergic (ephedrine) and β3-adrenergic (CL-316,243, etc.) agonists, have been unsuccessful in humans due to either undesirable cardiovascular effects, poor oral availability, or in the case of CL 316,243, weak agonism for the human β3-AR (69). Recently, a clinical study using a new class of β3-AR agonist increased energy expenditure via brown fat-meditated thermogenesis (70). Based on our review of the literature, the status of mitochondrial respiratory capacity may powerfully influence the outcome of β3-adrenergic agents and should be considered as adjunctive therapies to either restore or enhance the brown fat gene program.

## Concluding Remarks

**Figure 1:**
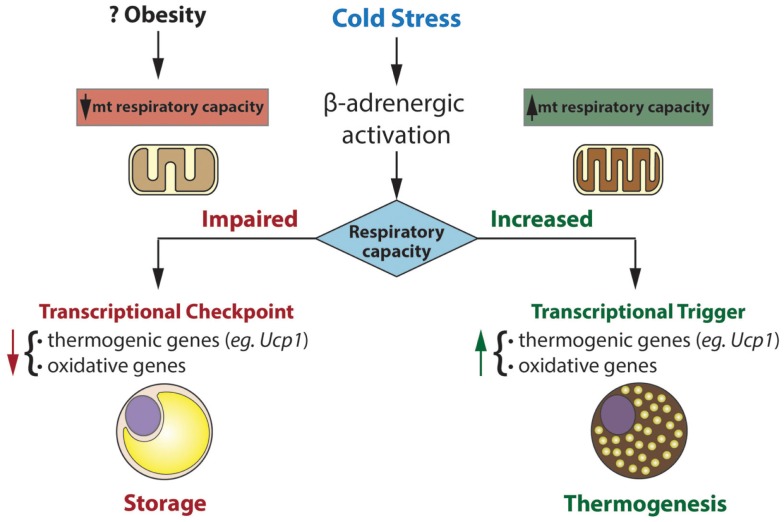
**Mitochondria exert transcriptional control over gene programs involved in oxidative metabolism and thermogenesis**. Transcriptional control is dependent on mitochondrial respiratory capacity. In this model, impaired respiratory capacity acts as a transcriptional checkpoint, whereas augmented respiratory capacity acts as a transcriptional trigger. In humans and mice, obesity is associated with reduced brown fat function. Our model may explain the inverse association between respiratory capacity and thermogenic gene expression in brown fat of certain obese mice (eg. *ob/ob*) and perhaps obese humans.

The putative role of mitochondrial respiratory capacity in transcriptional programs that regulate thermogenesis raises an intriguing question (Figure 1). Why is respiratory capacity *per se* linked to the regulation of the thermogenic gene program in brown fat? And what are the signals from mitochondria that govern this control? From a teleological perspective, impaired respiratory capacity would impair thermogenesis, making it a futile process. There seems to be interesting retrograde signaling from the mitochondrion to the nucleus, signaling, which may inactivate programs that expend energy and may promote storing that energy as lipid. Upon recovery of respiratory capacity, such signaling is turned off and energy expending programs are re-activated.

Control of nuclear genes based on the status of mitochondrial function (or stress) has been termed “mitohormesis” and is readily apparent in yeast and mammalian cells (71). In brown fat, a highly specialized tissue for thermogenesis, such mitohormesis may be crucial in matching functional and metabolic capacity to genetic programs that influence them.

In the future, it will be of great interest to unravel the signaling pathways by which respiratory capacity regulates the thermogenic gene program. Elucidating these signals and downstream pathways may inform the search for diagnostic and therapeutic interventions important for obesity and diabetes.

## Conflict of Interest Statement

The authors declare that the research was conducted in the absence of any commercial or financial relationships that could be construed as a potential conflict of interest.
